# Surgery-first segmental orthognathic surgery for treatment of hemimandibular elongation: a novel modified modality

**DOI:** 10.3389/fsurg.2026.1838908

**Published:** 2026-07-02

**Authors:** Jiawen Si, Yilang Du, Jingyang Huang, Hongbo Yu, Guofang Shen

**Affiliations:** Department of Oral and Craniomaxillofacial Surgery, Shanghai Ninth People’s Hospital, College of Stomatology, Shanghai Jiao Tong University School of Medicine, National Center for Stomatology, National Clinical Research Center for Oral Diseases, Shanghai Key Laboratory of Stomatology, Shanghai Research Institute of Stomatology, Shanghai, China

**Keywords:** hemimandibular elongation, mandibular asymmetry, mandibular body osteotomy, surgery-first, virtual surgical plan

## Abstract

**Background:**

The application of a surgery-first approach in patients with hemimandibular elongation (HE) remains poorly defined. In this pilot study, we introduce a modified surgery-first segmental orthognathic surgery (OGS) technique that integrates novel osteotomy design and structural precision to address HE.

**Methods:**

Nine patients with HE underwent segmental Le Fort I osteotomy, bilateral sagittal split ramus osteotomy (BSSRO), and unilateral mandibular body osteotomy, using both virtual surgical planning (VSP) and individualized cutting guides. Surgical accuracy was evaluated by comparing three-dimensional (3D) models obtained at 1 week postoperatively (T1) with VSP. Mid-term skeletal stability was assessed by comparing 3D models at T1 with those at 6–12 months postoperatively (T2). Symmetry-related parameters (chin deviation, chin rotation, bilateral mandibular body length discrepancy, and asymmetry index) were measured and compared.

**Results:**

All patients healed uneventfully without major complications. Surgical accuracy analysis showed a mean 3D deviation of 1.58 ± 0.39 mm between VSP and T1. Significant improvements were observed in all symmetry parameters at T1 (*p* < 0.05). At mid-term follow-up (mean 8.2 months, range 6–12 months), chin deviation, chin rotation, bilateral mandibular body length discrepancy, and asymmetry index showed no significant changes from T1 to T2.

**Conclusions:**

This pilot study demonstrates that the modified surgery-first segmental OGS technique achieves good surgical accuracy, immediate improvement in facial symmetry, and acceptable mid-term skeletal stability. This approach, integrating a unique osteotomy design, VSP, and 3D-printed cutting guides, offers a promising preliminary solution for complex facial asymmetry.

## 1 Introduction

Hemimandibular hyperactivity can be classified into three distinct pathological types according to Obwegeser and Makek's classification, namely: hemimandibular hyperplasia (HH), hemimandibular elongation (HE), and solitary condylar hyperplasia (1, 2). Clinically, HE is extremely rare and is characterized by horizontal elongation of the mandibular body on the affected side, chin deviation to the contralateral side, asymmetric dental malocclusion, and the absence of significant vertical hyperplasia of the ramus or condyle. Various treatment protocols have been proposed based on the severity of hemimandibular hyperactivity and patients’ requirements, such as orthognathic surgery (OGS) with orthodontic treatment, OGS with condylectomy, OGS with genioplasty, and mandibular contouring (2–6). Given the progressive deterioration of facial aesthetics and the prolonged waiting period required for the cessation of hemimandibular hyperactivity, patients with HE often prioritize immediate aesthetic improvement and shorter overall treatment duration over the sole correction of occlusal dysfunction (5, 7, 8). To date, however, the successful application of a surgery-first orthognathic approach specifically tailored to HE has not been reported, underscoring a critical gap in both surgical strategy and digital workflow integration.

With the rapid advancement of computer-assisted surgery (CAS) and patient-specific cutting guides, segmental osteotomies have become increasingly utilized in orthognathic surgery to enhance surgical outcomes. Huo et al. (9) fabricated a patient-specific cutting guide to pilot the endoscopically assisted vertical ramus osteotomy for the treatment of type 2 condylar hyperplasia. Haas et al. (10) introduced an intraoral proportional condylectomy guide for the treatment of condylar hyperplasia. Notably, our group has previously implemented various CAS techniques to refine surgical management in mandibular asymmetry and condylar hyperplasia, enabling more complex virtual surgical planning (VSP) and its accurate translation to the operating room (3, 11, 12).

In line with these developments, the present study introduces a modified surgery-first approach (SFA) for the treatment of HE that combines segmental Le Fort I osteotomy, BSSRO, and unilateral mandibular body osteotomy facilitated by VSP and individualized cutting guides. The integrated workflow is designed to achieve several key objectives: establishing normalized overjet, overbite, and dental midline; creating a decompensated and stable molar relationship; coordinating maxillary and mandibular arch forms; and correcting jaw asymmetry. By reporting on surgical accuracy, effectiveness, and complication profiles, this study provides proof-of-concept for a novel surgical paradigm for the correction of complex facial asymmetry that integrates a unique osteotomy design and structural precision.

## 2 Materials and methods

**Figure 1:**
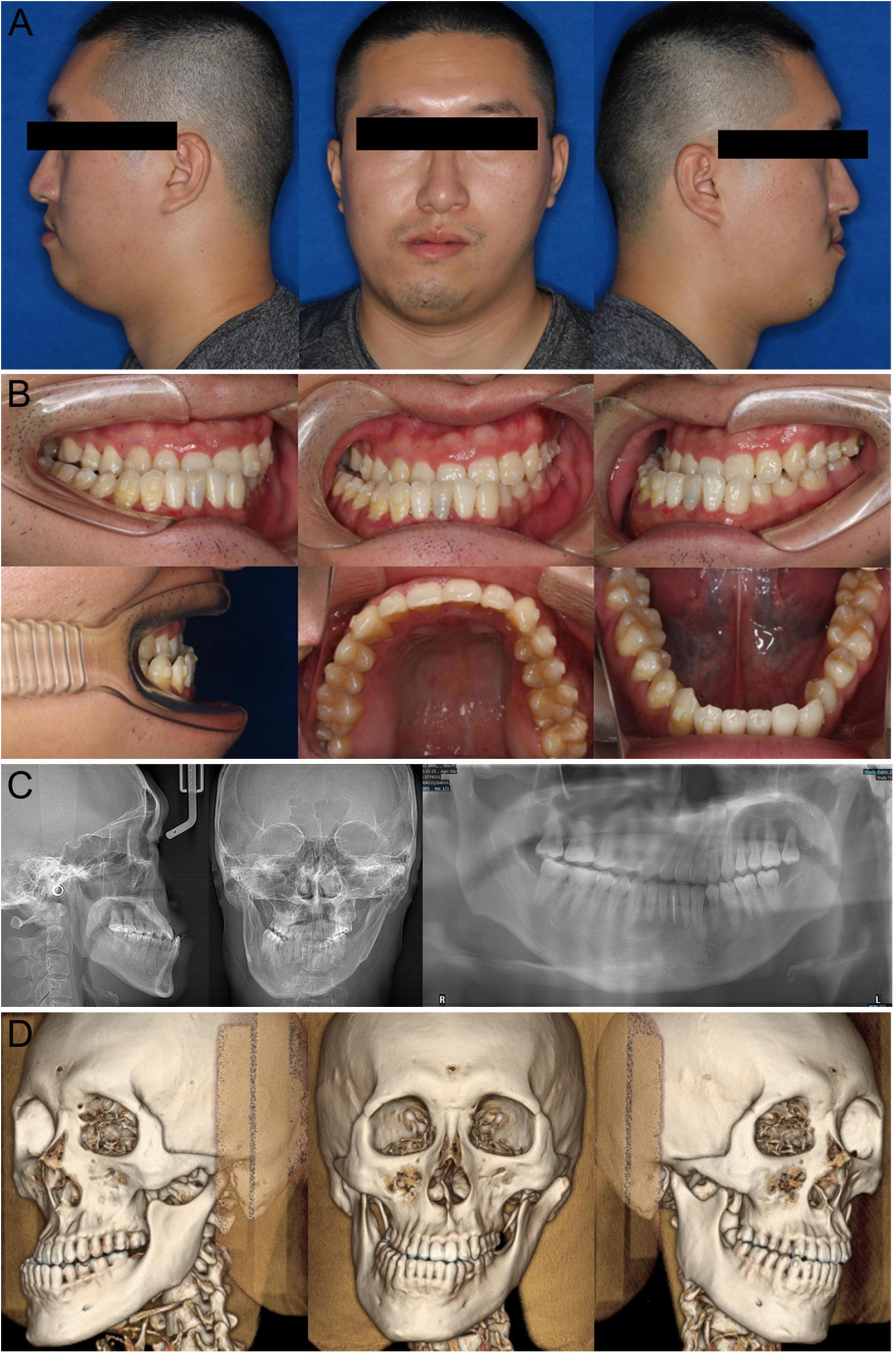
Representative patient with a chief complaint of facial asymmetry. **(A)** Pretreatment facial photographs show severe hyperplasia of the left mandibular body and chin deviation 15 mm to the right; **(B)** intraoral photos show labially inclined upper incisors, deviated lower dental midline to the right and right posterior teeth buccally crossbite; **(C)** pretreatment lateral and posteroanterior cephalograms as well as panoramic radiographs; **(D)** pretreatment CT scan shows right deviation of the chin and left mandible body significantly lengthened horizontally.

This retrospective study was conducted at the Department of Oral and Craniomaxillofacial Surgery, Shanghai Ninth People's Hospital, and was approved by the institutional ethics committee (SH9H-2019-T114-1). From January 2020 to December 2024, nine patients with the diagnosis of unilateral HE were included in this study (Figure 1).

The inclusion criteria were as follows: (1) clinical and radiographic (CT and panoramic X-ray) diagnosis of unilateral HE based on the Obwegeser and Makek's classification as previously reported (1, 2); (2) confirmation of inactive condylar growth via preoperative single-photon emission computed tomography (SPECT) using an optimal cut-off reference value of 13% for evaluating hemimandibular hyperplasia in Chinese patients, as previously described (3, 13); (3) mild dental crowding (Little's Irregularity Index ≤4 mm); (4) age ≥18 years; and (5) complete preoperative, intraoperative and postoperative data collection and informed consent throughout the surgical procedure and follow-up process.

Exclusion criteria are as follows: (1) Active condylar hyperplasia confirmed by SPECT; (2) prior pre-surgical orthodontic decompensation performed; (3) history of prior orthognathic surgery or maxillofacial trauma; (4) uncontrolled temporomandibular joint pathology; (5) congenital craniofacial anomalies (e.g., cleft lip/palate, hemifacial microsomia); and (6) other systemic conditions that may affect the results of orthognathic surgery.

All patients underwent surgery: first, a segmental Le Fort I osteotomy, BSSRO, and unilateral mandibular body osteotomy aided by both VSP and individualized cutting guides. The postoperative orthodontic treatment protocol of the patients was conducted as we and other literature reported previously (4, 6). Briefly, after patient consultation and examination by the surgeon and orthodontist, suitability for SFA was determined. No active pre-surgical decompensation was performed in any patients. Treatment commenced at 2–4 weeks after surgery using fixed 0.022-inch pre-adjusted edgewise appliances. The intended transitional occlusion (ITM) splint, manufactured on the basis of VSP, was used as a guide for bite settling. Active orthodontic tooth movement was facilitated by the regional acceleratory phenomenon evoked by the surgery.

### 2.1 Computer-assisted surgical planning and cutting guide 3D-printing

The VSP and cutting guide 3D-printing were performed as previously reported by our group (3). Briefly, DICOM data files obtained from maxillofacial computed tomography (CT; LightSpeed CT scanner, GE Healthcare, UK) were imported into the ProPlan CMF software (Materialise, Belgium), and the segmental Le Fort I osteotomy, BSSRO, and unilateral mandibular body osteotomy were designed. The osteotomy line for the unilateral mandibular body osteotomy the planned was delineated virtually based on multiplanar (axial, coronal, and sagittal) and three-dimensional (3D) views. The data sets of the bony segments after the osteotomy and occlusions were virtually imported into Geomagic Studio 2013 software (Geomagic, USA) to design and 3D-print the cutting guides and splint.

### 2.2 Surgical procedure

**Figure 2:**
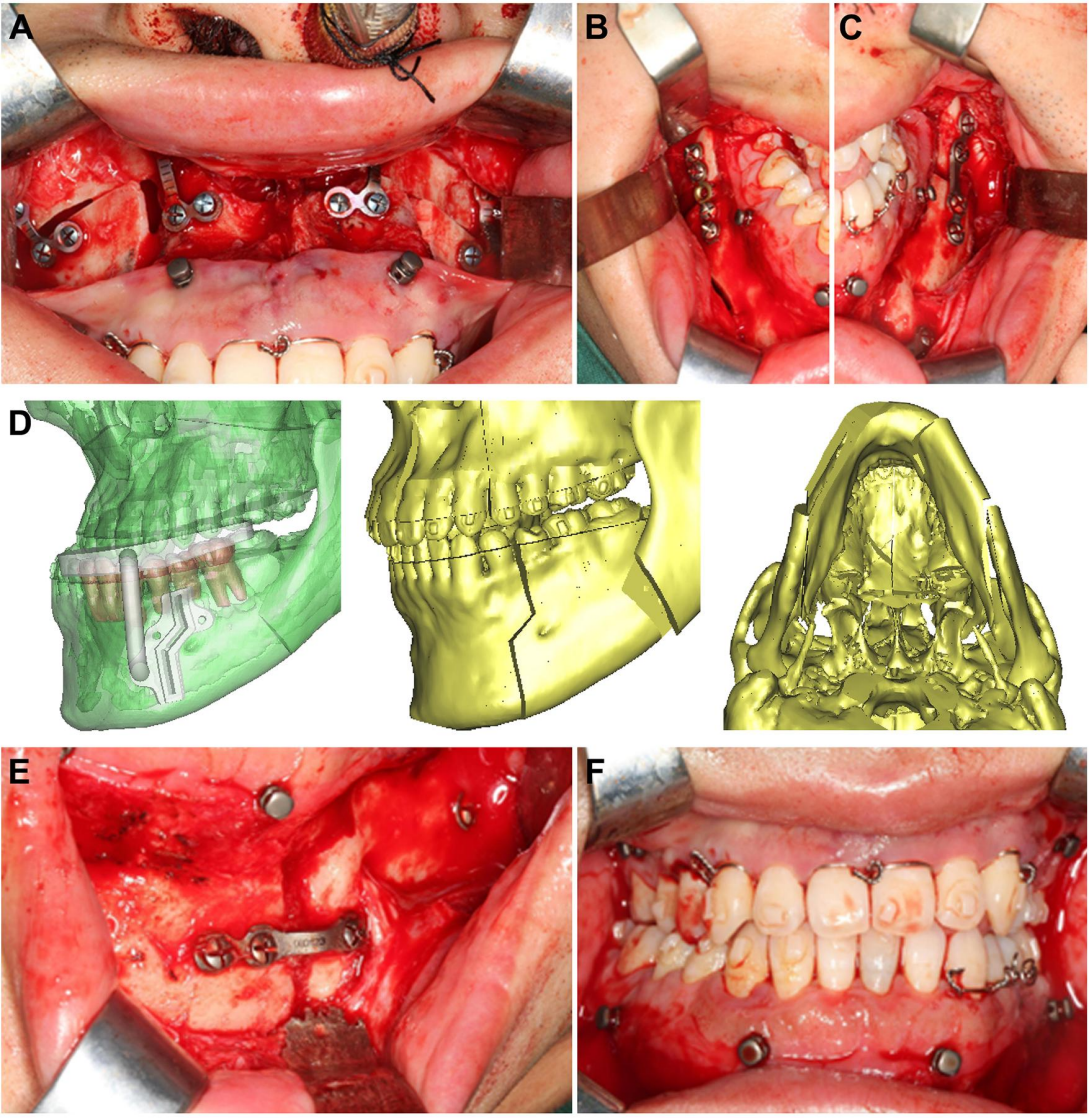
Representative intraoperative photos and schematic VSP for left mandibular body osteotomy. **(A)** Segmental Lefort I osteotomy into four pieces is performed to correct the secondary maxillary deformity; **(B, C)** bilateral sagittal split ramus osteotomy; **(D)** schematic VSP for individualized cutting guide aided left mandibular body osteotomy, note the stepped osteotomy line was designed to avoid trauma to adjacent teeth root and IAN; **(E)** left mandibular body osteotomy between the lower left first and second premolar; **(F)** intraoperative occlusion.

**Figure 3:**
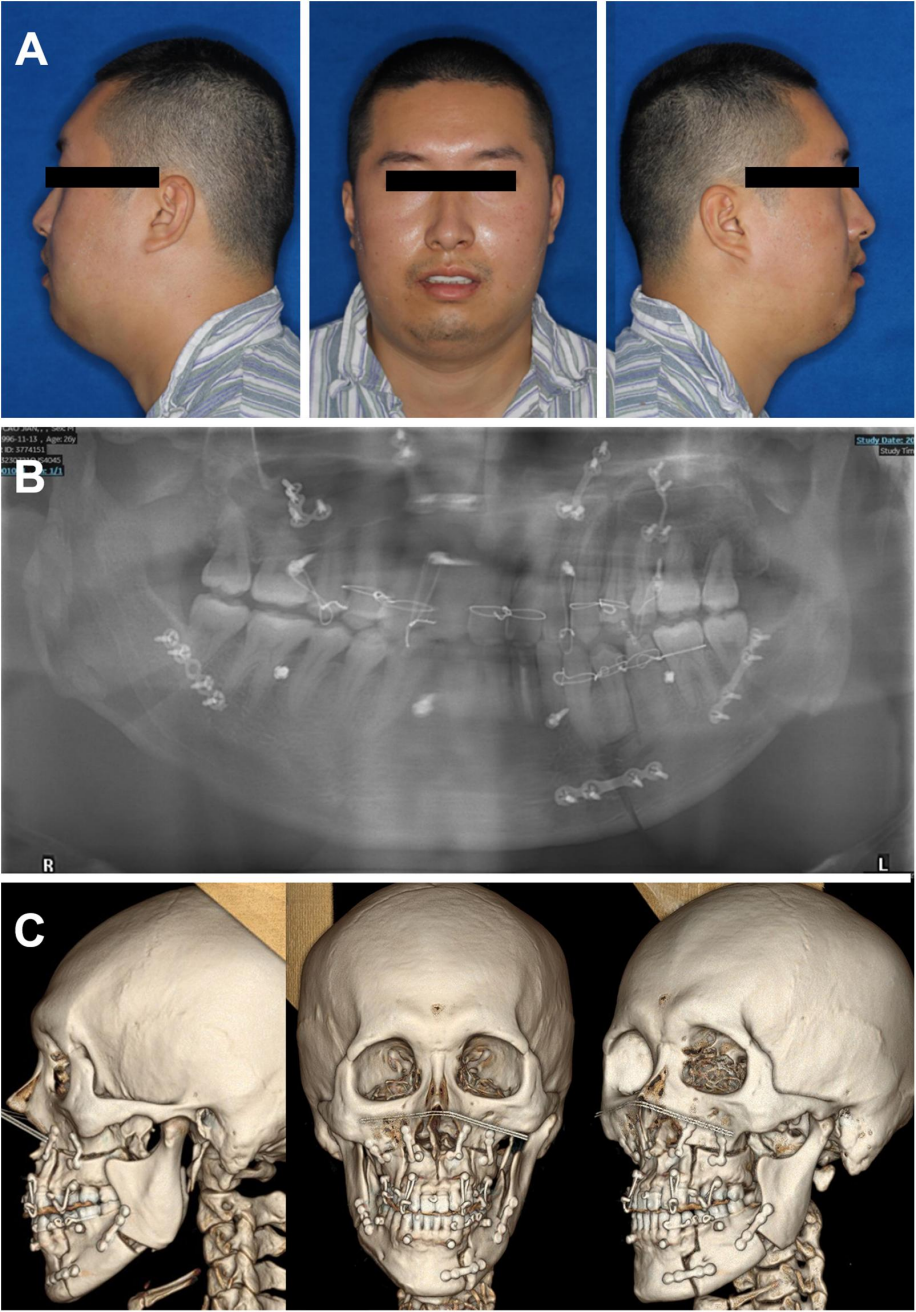
Representative patient at 3 days after the surgery (T1). **(A)** Postoperative photographs show the significant improvement of facial asymmetry; **(B)** postoperative panoramic radiograph indicates no dental root injury near the segmental osteotomy lines and Class I canine and molar relationships are attained; **(C)** postoperative CT scan shows the left hyperplastic mandible is now similar in appearance to the right side and all segments was fixed with the use of titanium mini-plates and screws as well as an ITM splint.

All operations were performed by the authors' surgical team. Briefly, patients underwent a segmental Le Fort I osteotomy, BSSRO, and unilateral mandibular body osteotomy according to the VSP (Figure 2). Additional contour trimming was performed in two cases to optimize the esthetic outcomes. For the template-guided mandibular body osteotomy, anatomic landmarks of the mandibular body were identified, and the individualized osteotomy guide covering the buccal border of the mandibular body was positioned and fixed with temporary screws according to its surface best fit. Then, a step osteotomy at the level of the cutting plane was performed, successfully avoiding trauma to adjacent tooth roots and nerves (Figure 2D,E). After surgery, the ITM occlusal splint was fixed on the upper teeth, and light elastic wear was used to achieve a stable initial occlusion and jaw position (Figure 3).

### 2.3 Surgical accuracy evaluation

**Figure 4:**
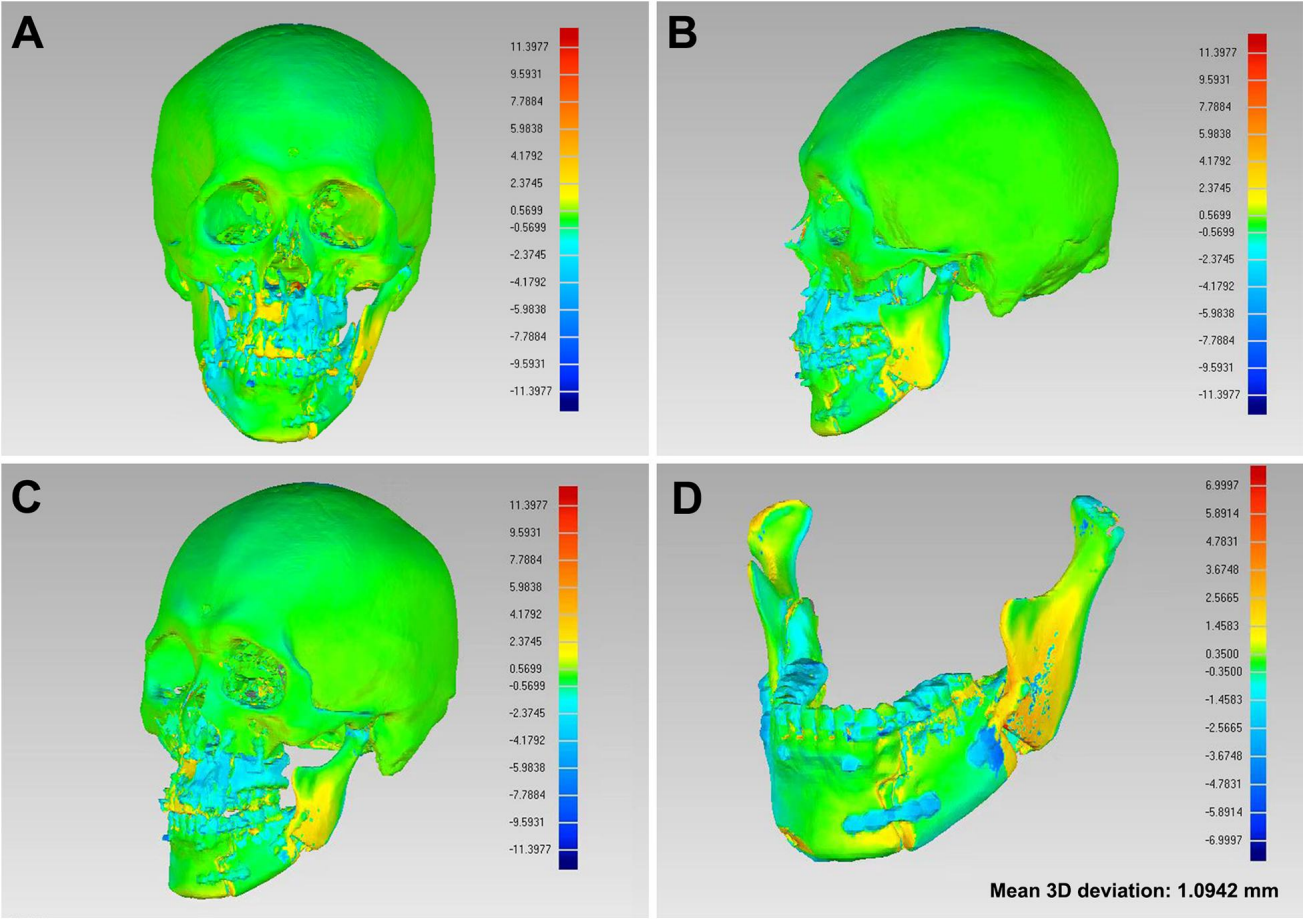
The 3D models of the virtual surgical plan and the CT scan of the actually achieved outcome were reconstructed to validate the surgical accuracy. **(A)** Frontal view; **(B)** lateral view; **(C)** oblique view; **(D)** detailed view of the mandibular body osteotomized area and mean 3D deviation of the mandible.

The evaluation of surgical accuracy was performed as previously described (3, 14). Briefly, a craniomaxillofacial CT scan was obtained for each patient three days postoperatively (T1). The 3D models of the virtually planned and actual postoperative outcomes were reconstructed and superimposed using ProPlan CMF software (Materialise, Belgium). The superimposed STL files were then imported into Geomagic Studio 2013 software (Geomagic, USA), and the deviations of the mandible were measured as the mean 3D deviation (Figure 4).

### 2.4 Surgical effectiveness evaluation

The ProPlan CMF software (Materialise, Belgium) was used to reconstruct 3D virtual models and evaluate mandibular symmetry preoperatively, virtually (VSP), and postoperatively, as previously described (3, 13). Briefly, several landmark points, namely nasion (N), bilateral orbitale (OrL, OrR), bilateral condylar tip (CoL, CoR), bilateral porion (PoL, PoR), basion (Ba), pogonion (Pog), menton (Me), bilateral mental foramen (MFL, MFR), sigmoid notch (Sg), and bilateral gonions (GoL, GoR) were marked on the virtual craniofacial skeleton model. Additionally, a landmark GoM was defined at the midpoint of the GoL–GoR line. Then, three reference planes and the basic coordinate system (x, y, z), with the N point as the origin, were established. Moreover, the mandibular plane was defined as a plane passing through Me, GoL, and GoR, while the mandibular midsagittal plane was defined as a plane that was perpendicular to the mandibular plane and passing through GoM and Me. Finally, several measurements were calculated, namely the discrepancy of bilateral Go-Pog (the discrepancy between bilateral mandibular body lengths), chin deviation (the distance from the Pog to the mandibular midsagittal plane), chin rotation (the angle between the facial midsagittal plane and the mandibular midsagittal plane), and the asymmetry index (AI) as previously reported (3).

### 2.5 Surgical complications and mid-term skeletal stability assessment

All patients were followed for 6–12 months. A preliminary assessment of surgical complications was conducted during the follow-up period. Briefly, surgical complications were assessed based on clinical examination for malocclusion relapse, mental nerve injury, postoperative bleeding, and prolonged or none bone-union. The inferior alveolar nerve (IAN) injury was evaluated using a light-touch sensation examination, as previously described (15). To evaluate mid-term mandibular skeletal stability, 3D CT scans were obtained at the last postoperative follow-up (T2). The same 3D measurement protocol used for evaluating surgical accuracy was applied to assess changes from T1 to T2. The 3D positional change of the mandibular Pog point within the established coordinate system at T2 was calculated to assess mandibular relapse.

### 2.6 Statistical analysis

GraphPad Prism software (GraphPad Software, CA) was used for statistical analysis and graphical representation. Normalized measurement data are presented as the mean ± standard deviation. Numerical data comparisons were performed using dependent or independent *t*-tests, where *P* < 0.05 was considered statistically significant.

## 3 Results

A total of nine patients (five males, four females; mean age 22.4 ± 2.7 years, range 19–25 years) were included in this study, and their clinical information is presented in Table 1. After superimposing the VSP and the 3D models of the postoperative CT scans at T1, the osteotomy deviation analysis showed that the surgical outcome exhibited a high degree of similarity to the VSP (Figure 4). In detail, the mean 3D deviation of the postoperative mandible at 3 days postoperatively compared with the VSP was 1.58 ± 0.39 mm (Table 1), indicating that the correction of mandibular symmetry was performed with good accuracy.

**Table 1:** The patients' clinical information, mean 3D deviation and surgical complication.

No.	Age	Sex	Hyperplastic side	Surgery mean 3D deviation of the mandible	Surgical complication
Patient 1	25	Male	Left	1.0942	None
Patient 2	23	Male	Right	1.2092	None
Patient 3	22	Female	Left	1.1313	None
Patient 4	24	Female	Left	1.8623	None
Patient 5	20	Female	Right	1.8519	None
Patient 6	23	Female	Left	1.9004	None
Patient 7	19	Male	Left	1.5136	None
Patient 8	23	Male	Left	1.4675	None
Patient 9	21	Female	Right	2.1763	Bad split on R mandible^a^

a Bad split was not severe and healed uneventfully without additional management.

**Figure 5:**
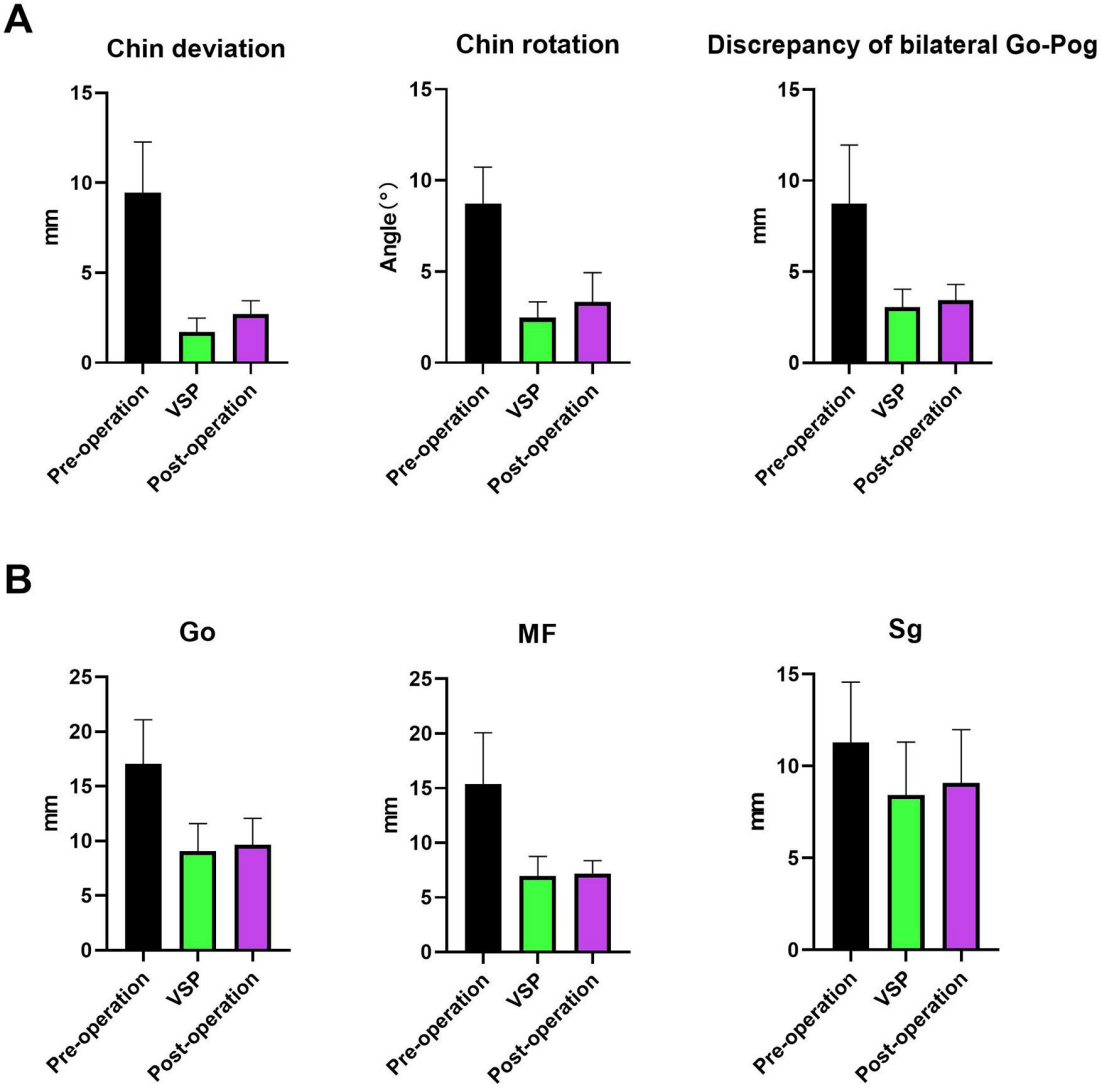
The preoperative, VSP, and postoperative mandibular symmetry assessment. **(A)** the preoperative, VSP, and postoperative chin deviation, chin rotation, and discrepancies between bilateral Go-Pog; **(B)** the preoperative, VSP, and postoperative asymmetry index of the landmarks Go, MF, and Sg.

In terms of the surgical effectiveness, the discrepancy between bilateral mandibular body lengths was significantly reduced by the combination of BSSRO and unilateral mandibular body osteotomy (from 8.73 ± 3.22 to 3.44 ± 0.87 mm, *P* < 0.05), while the chin deviation, chin rotation, and AI all showed significant postoperative improvements (Figure 5). Chin deviation was significantly decreased postoperatively (from 9.45 ± 2.82 to 2.70 ± 0.74 mm, *P* < 0.05), while the angle of chin rotation was significantly reduced postoperatively (from 8.73 ± 2 to 3.33 ± 1.61°, *P* < 0.05). The asymmetry indices of the landmarks Go and MF decreased significantly (AI of Go: from 17.07 ± 4.01 to 9.64 ± 2.44 mm, *P* < 0.05; AI of MF: from 15.36 ± 4.72 to 7.20 ± 1.17 mm, *P* < 0.05; AI of Sg: from 11.29 ± 3.28 to 9.10 ± 2.88 mm).

**Figure 6:**
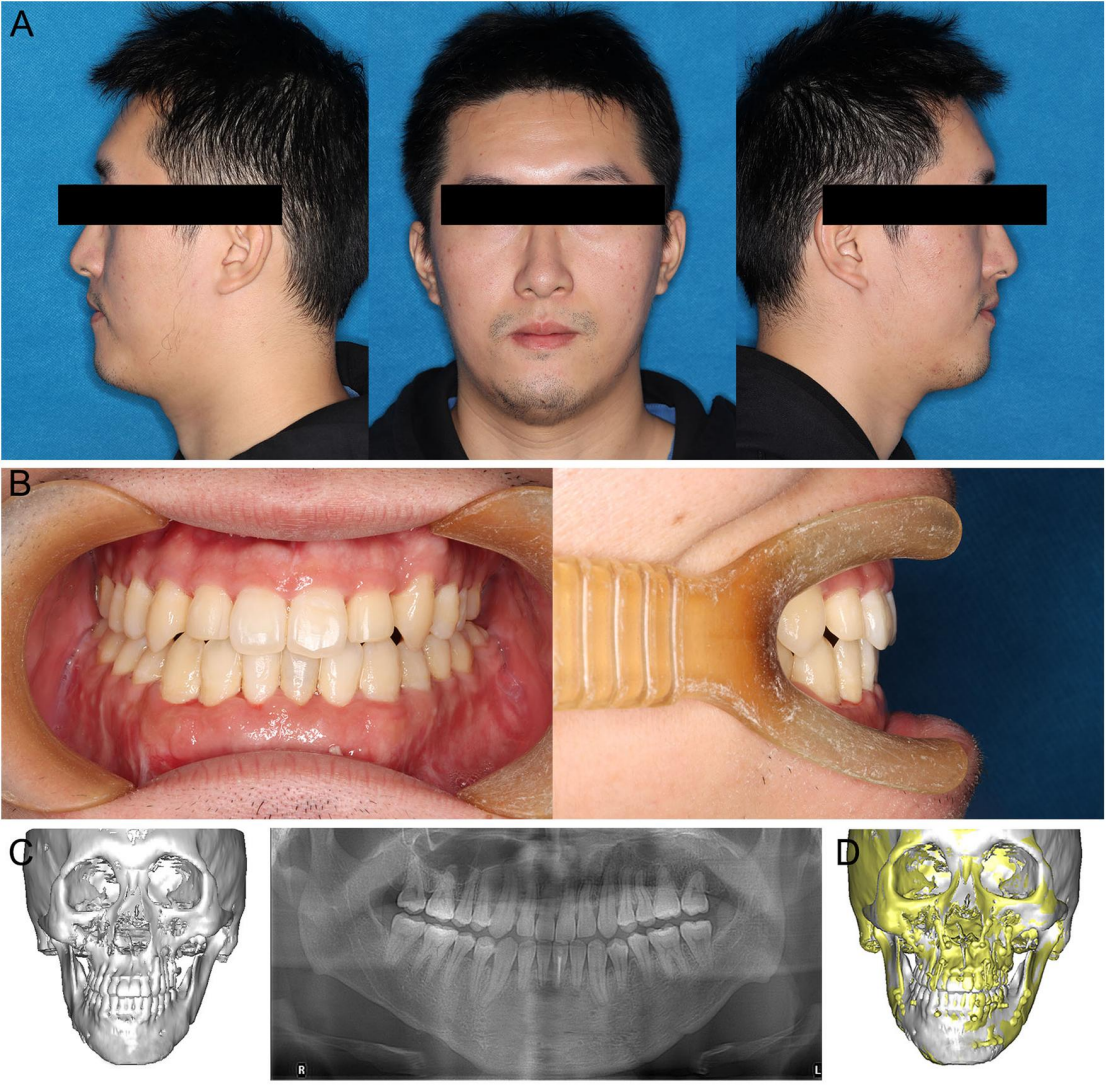
Representative patient at 12 months after the surgery (T2). **(A)** Follow-up photographs show the well-maintained facial symmetry; **(B)** follow-up photographs show the well-maintained Class I canine and molar relationships; **(C)** CT scan and x-ray at T2 show proper healing of all segments; **(D)** merge of 3D models of CT scan at T1 (yellow) and T2 (white).

The mean follow-up duration for the nine patients was 8.2 months (range: 6–12 months). Although one patient presented with a minor bad split, he healed uneventfully without additional management. No severe malocclusion relapse, postoperative bleeding, infections, permanent IAN neurosensory disturbance, none bone-union, or skeletal instability were noted during follow-up (Figure 6). To evaluate the mid-term skeletal stability, mandibular parameters from T1 to T2 were assessed and summarized in Table 2. Briefly, the chin deviation (increased from 2.70 ± 0.74 mm to 3.15 ± 0.92 mm), chin rotation (increased from 3.33 ± 1.61° to 3.58 ± 1.70°), bilateral mandibular body length discrepancy (increase from 3.44 ± 0.87 mm to 3.62 ± 1.04 mm), and asymmetry indices (AI of Go, AI of MF, AI of Sg) showed minimal, nonsignificant increases from T1 to T2 (all *P* > 0.05), indicating that the surgical results of the mandible were largely maintained over the 6–12 months period. Moreover, the 3D position of the mandibular Pog point at T2 showed forward translation of 1.52 ± 1.21 mm, upward translation of 1.08 ± 1.35 mm, and lateral translation of 1.32 ± 0.41 mm, respectively.

**Table 2:** Postoperative skeletal changes from 3 days after surgery (T1) to mid-term follow-up (T2).

Parameter	T1	T2	Change (T2–T1)	*p*-value
Chin deviation (mm)	2.70 ± 0.74	3.15 ± 0.92	+0.45 ± 0.61	0.082
Chin rotation (°)	3.33 ± 1.61	3.58 ± 1.70	+0.25 ± 0.55	0.214
Bilateral mandibular body length discrepancy (mm)	3.44 ± 0.87	3.62 ± 1.04	+0.18 ± 0.70	0.462
AI of gonion	9.64 ± 2.44	10.12 ± 2.68	+0.48 ± 0.89	0.147
AI of mental foramen	7.20 ± 1.17	7.65 ± 1.35	+0.45 ± 0.71	0.093
AI of sigmoid notch	9.10 ± 2.88	9.50 ± 3.05	+0.40 ± 0.95	0.215

## 4 Discussion

The severe facial asymmetry and malocclusion caused by hemimandibular hyperactivity can only be corrected with the combined treatment of orthodontics and orthognathic surgery (2, 7, 16). Due to significant deterioration in facial appearance and the long waiting time before hemimandibular hyperactivity resolves, these patients have a greater demand for immediate facial aesthetic improvement and a shorter overall treatment duration than for correcting occlusal dysfunction (5, 7, 8). Thus, the concept of the SFA was proposed (17–20).

To date, the feasibility of SFA has been demonstrated in Class II and Class III malocclusion cases; however, the proper use of SFA for facial asymmetry and lateroprognathism is still unclear (18–21). Guo et al. (7) assessed the feasibility of SFA in 12 patients with facial asymmetry by measuring mandibular height, maxillary height, mandibular body length, and ramus length. No difference in these parameters between the symmetric and asymmetric sides was noted during follow-up. Choi et al. (4) also assessed the treatment efficacy of SFA in skeletal dentofacial asymmetry and found similar results by measuring posteroanterior cephalometric radiographs. In this pilot study, we introduce, for the first time, the successful application of surgery-first segmental Le Fort I osteotomy, BSSRO, and unilateral mandibular body osteotomy for the treatment of unique HE cases with immediate improvements across all symmetry parameters. At mid-term follow-up, the chin deviation, chin rotation, bilateral mandibular body length discrepancy, and asymmetry index showed no significant changes from T1 to T2. Our findings align with previous analyses of SFA orthognathic efficacy and stability in improving skeletal symmetry. Notably, the minimal lateral relapse in chin deviation and the AI of Go were possibly due to the additional stability provided by the unilateral mandibular body osteotomy. Moreover, Atipatyakul et al. (5) prospectively evaluated 70 Class III asymmetry patients treated with SFA and reported a mandibular distal segment relapse at 1 year (forward: 1.9 ± 1.6 mm; upward: 1.1 ± 1.5 mm), while our observations showed a mandibular distal segment forward translation of 1.52 ± 1.21 mm, upward translation of 1.08 ± 1.35 mm and lateral translation of 1.32 ± 0.41 mm, respectively. This consistency suggests that the mild relapse observed in our cohort of HE cases is not unique to the segmental osteotomy technique but rather reflects the inherent stability profile of SFA for asymmetry correction (22).

Over the last decade, indications for SFA have been proposed in the literature, evolving as surgical and CAS techniques have improved. According to early criteria for SFA, an increase in the number and complexity of osteotomy procedures poses a greater risk of increased complications and potential challenges in planning and predicting outcomes (23, 24). However, when there is an arch width discrepancy, an asymmetric transverse arch, or severe facial asymmetry, it is difficult to simulate the possible orthodontic movements and achieve a favorable outcome without segmental osteotomies (24–26). Notably, Yorikatsu et al. (27) introduced a surgery-first segmental Le Fort I osteotomy, followed by mandibular osteotomy and distraction osteogenesis, for treatment of severe facial asymmetry. Their method has been successfully used in five patients with severe latero-retrognathism, supporting the feasibility of complex segmental osteotomies for facial asymmetry within a surgery-first approach. Likewise, our modified technique, designed for the treatment of HE, helps to widen the indications for SFA in severe facial asymmetric deformities.

Particularly, compared with the previously reported surgery-first double-jaw approach, we modified the surgical plan to include a segmental Le Fort I osteotomy, BSSRO, and a unilateral mandibular body osteotomy. In typical HE, horizontal hyperplasia of the mandible pushes the chin toward the unaffected side, while the mandibular bodies on both sides lie on the same level. The affected side of the mandibular body is significantly lengthened horizontally, appearing relatively straight rather than bowed, compared with the unaffected side. The lower dentoalveolar arch is also bent with an asymmetrical contour, the lower dental midline drifts to the unaffected side, the anterior teeth exhibit a deformed axial inclination to the basal bone, and the contralateral posterior teeth cross buccally (2, 28). Thus, the overall surgical treatment goals of SFA in HE cases include obtaining relatively normal overjet, overbite, and dental midline, establishing a decompensated stable molar relationship, coordinating the maxillary and mandibular arch forms, and correcting jaw asymmetry. From a clinical perspective, we believe the modified surgery-first techniques reported in this study are more feasible to achieve several treatment goals in HE cases. First, the segmental maxillary Le Fort I osteotomy into multiple pieces, BSSRO, and unilateral mandibular body osteotomy provided an immediate means to realign the teeth, coordinate the upper and lower dental arches, and establish a more stable surgical temporary occlusion. Additionally, the inclination of upper incisors, bilateral molar relationship, and curve of Spee of the ITM can be established with segmental osteotomies by manipulating the position and rotation of different dentoalveolar segments, which is utilized as a starting point to guide postoperative orthodontic treatment (23). Moreover, the BSSRO and unilateral mandibular body osteotomy help to rebuild the dentoalveolar arch contour and reduce mandibular asymmetry. Lastly, the SFA should be performed based on a delicate and precise VSP and close cooperation among the orthognathic surgeon, orthodontist, and CAS engineers, which is mandatory for achieving predictability and a favorable outcome (4). By using a cutting guide aligned and fixed on the buccal side of the mandibular body, the mandibular body osteotomy can be performed with adequate space and safe access, thus avoiding the risk of injuring the dental roots and IAN (Figure 2D) (9, 14, 29, 30).

Nevertheless, this study has several limitations that must be acknowledged. With only nine patients and a mid-term follow-up period, this pilot study is statistically underpowered. As such, it should be considered a pilot case study. Larger prospective studies with long-term follow-up periods beyond 1 year are needed to validate our findings. Moreover, because we were unable to record changes in dental conditions and occlusion during the follow-up period, this study did not include comprehensive orthodontic treatment outcomes and occlusal evaluations (e.g., final overjet/overbite, angle classification, periodontal health, or root resorption assessment) due to its retrospective nature. As occlusal stability and dental health are critical outcomes of orthognathic surgery, evaluations of the overall treatment stability, as well as standardized dental assessments, should be performed in future prospective studies with larger sample sizes and longer follow-up intervals. Finally, our technique is limited to patients with HE with mild dental crowding (Little's Irregularity Index ≤4 mm), whereas patients with significant crowding or arch width discrepancies may not be suitable for this approach. Despite these limitations, this study provides the first detailed report of a surgery-first segmental protocol specifically tailored to HE, with both immediate accuracy and mid-term stability data.

## 5 Conclusion

This pilot study presents a novel modified surgery-first segmental orthognathic technique for HE that integrates VSP, patient-specific cutting guides, and a unique osteotomy design, achieving high precision and surgical efficiency without severe complications or mid-term mandibular skeletal instability. Despite the limitations of a small sample size and lack of detailed occlusal outcome data, this approach offers a promising preliminary solution for the predictable, digitally driven correction of complex facial asymmetry and exemplifies a new paradigm of structural precision and data-integrated craniofacial surgery. Larger prospective studies with longer follow-up periods and comprehensive dental outcome assessments are warranted to confirm long-term stability and treatment efficacy.

## Data Availability

The original contributions presented in the study are included in the article, further inquiries can be directed to the corresponding author/s.
